# Regulation of adaptive immune responses by guiding cell movements in the spleen

**DOI:** 10.3389/fmicb.2015.00645

**Published:** 2015-06-25

**Authors:** Lintao Zhao, Lina Liu, Bo Guo, Bo Zhu

**Affiliations:** ^1^Institution of Oncology, Xinqiao Hospital, Third Military Medical UniversityChongqing, China; ^2^Department of Microbiology, Third Military Medical UniversityChongqing, China

**Keywords:** spleen, cell migration, immunoregulation, T cells, CCL21

## Abstract

The spleen combines the innate and adaptive immune systems in a uniquely organized way. The excision of spleen will induce many complications, especially the increased susceptibility to infections. Recent research shows that besides playing roles during the immune responses, the spleen is also an important organ during immunoregulation, which is different from other secondary lymphoid organs. This unique function is mainly realized by modulating cell migration and proliferation in the spleen. This review provides a better understanding of the functions of this complex organ gained from recent studies.

## Introduction

The adaptive immune responses play important roles in anti-infection *in vivo* and in clearance of exogenous pathogens. The reactivation of these processes necessitates the contact between antigen-specific T/B lymphocytes and antigen-presenting cells carrying corresponding antigens. However, such contact occurs very rarely *in vivo* (Gretz et al., 1996; Bajenoff et al., 2006; Cesta, 2006) and should be realized in secondary lymphoid organs (SLOs). The spleen is a very important SLO. On a daily basis, a larger count of lymphocytes passes through the spleen than the total count passing through all other SLOs (Bajenoff et al., 2008). The earliest research shows that the excision of the spleen will not largely impact the human immune system. However, recent research shows that this excision will induce many complications, especially the increased susceptibility to infections. These studies indicate that the spleen plays exclusive roles that cannot be fulfilled by other SLOs (Cadili and de Gara, 2008). Moreover, besides playing roles during immune responses, the spleen is also an important organ during immunoregulation, which is different from other SLOs. The spleen could decrease the probability of contact between antigen-specific T/B lymphocytes and antigen-presenting cells carrying corresponding antigens. In addition, it could also induce the extensive accumulation of immunosuppressive cells. Moreover, this characteristic is unique to the spleen among all SLOs. This review focuses on the advances in recent research on the spleen.

## Structure of the Spleen

### White Pulp and Red Pulp

**FIGURE 1:**
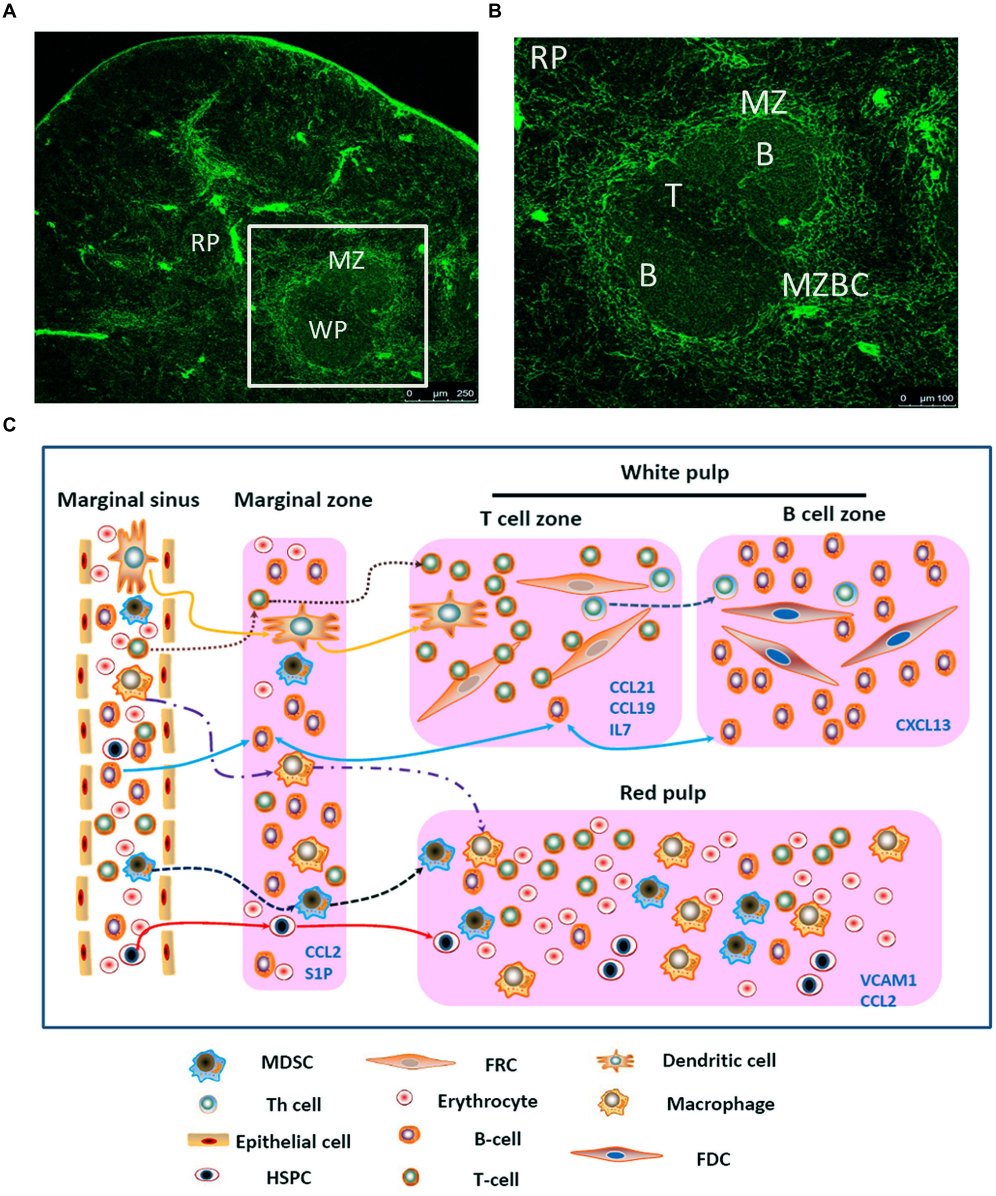
**Migration of different types of cells in the spleen. (A)** Spleen cryostat sections from C57BL/6 mice were stained for ER-TR7 (green), and imaged using confocal microscopy (Scale bars: 250 μm); **(B)** a higher magnification of spleen cryostat sections were observed; **(C)** different cell components in the blood enter the marginal sinus, and diffuse to the marginal zone. T and B cells will first arrive at the T cell zone. Then follicular Th cells and B cells would move further to the B cell zone. Moreover, B cells also could shuttle continually between the MZ and follicles. The dendritic cells, after reactivation, could enter the T cell zone. MDSCs could be attracted by CCL2 and retained in the MZ and red pulp. Besides, adhesion molecule VCAM-1in red pulp could retain bone marrow HSPCs.

The spleen is composed of white pulp and red pulp. The white pulp consists of T and B lymphocytes, which reside in the T-cell zone and the B-cell zone, respectively, rather than mixing together (**Figures 1A,B**). Besides T cells, the T-cell zone is filled with mesenchymal cells, mainly fibroblastic reticular cells (FRCs), while the mesenchymal components of the B-cell zone are mainly fibroblastic dendritic cells (FDCs; Turley et al., 2010; den Haan et al., 2012). In addition, the red pulp which surrounds the white pulp is a loose web-like structure constituted by fibrous tissues. The aging red blood cells (RBCs) will be cleared away from this structure. The spleen with rich blood supply is an organ constituted by blood vessels encircling different branches. The blood after entering the spleen will pass through different levels of artery branch vessels. Finally, a part of the blood mingles into the marginal sinus (between the marginal zone and the white pulp); the other part directly enters the blood sinus in the red pulp and finally flows outside the spleen through the splenic veins. Specifically, the lymphocytes after entering the marginal sinus can directionally migrate to the surroundings of the central artery in the arterial terminal branches (Mebius and Kraal, 2005; Cesta, 2006) to form white pulp. On the contrary, other cell components will flow along with the blood into the red pulp. The red pulp contains abundant phagocytes, which will clear away the old RBCs and the pathogenic microorganism (den Haan et al., 2012).

### The Marginal Zone

This zone resides between the splenic white pulp and red pulp. This structure is unique among all splenic SLOs (**Figure 1B**). In this zone, abundant macrophages termed marginal-zone macrophages and marginal-zone metallophilic macrophages (MMMs), as well as dendritic cells (DCs) designated as marginal-zone DCs, and B cells (Bajenoff et al., 2008) pathogenic microorganism and aging cells will be largely swallowed by the macrophages, while the DCs will ingest the antigens. Moreover, the lymphocytes flowing into the blood will stay for a short period before entering the white pulp. The lymphocyte and the reactivated antigen-presenting cells, after arriving at the marginal zone, will enter the white pulp along specific routes, rather than randomly. In the MOMA-1-positive marginal zone, the macrophages will constitute a ring-like structure surrounding the white pulp, which helps to fully isolate the white pulp and the marginal zone. However, this ring-like structure is not perfect, since it has a gap. These cells are called marginal-zone B cells (MZBCs). This gap is a channel where various cells enter the white pulp. Interestingly, MZBCs only exist around the T-cell zone in the white pulp, rather than the B-cell zone (Bajenoff et al., 2008). These facts increase the probability of contact between the antigen-specific T/B lymphocytes and the antigen- presenting cells carrying corresponding antigens. For example, the antigen-presenting cells after activation would migrate to in the T-cell zone and retain there. The antigen-specific T cells migrate along the specific track into and out of the T-cell zone. These abilities allow T cells to move throughout the entire T-cell zone. As a result, the probability of contact between the antigen-specific T lymphocytes and the antigen- presenting cells carrying corresponding antigens could be maximized. On the contrary, this probability would be reduced greatly if the T cells can migrate into and out of the splenic T-cell zone randomly. Moreover, the marginal zone also contains a specific B cell subzone. MZBCs which reside between the marginal zone and the red pulp can capture antigens carried in the blood via complement receptors, and promote both T-cell-independent and -dependent immune responses. <article-title>

### Conduit System

There is a special harrow 3D web-like structure in the SLOs, which is constituted by FRCs as well as the FRCs-secreted extracellular matrix (ECM).</article-title> These FRCs mainly express podoplanin (gp38), but not the lymphatic and blood endothelial cell marker: platelet endothelial cell adhesion molecule (PECAM, CD31; Mueller and Germain, 2009; Malhotra et al., 2013). The FRCs wrap up a harrow microtubule system, constituted by the FRCs-secreted ECMs. The microtubule system in the SLOs can be detected by ER-TR7 (Alvarenga and Marti, 2014). The diameter of the microtubules is usually 4–5 nm, which are only accessible to molecules smaller than 70 KD. The distributive characteristics of these microtubules in the lymph nodes (LNs) have been thoroughly studied, but those in the spleen are unclear. It is deduced that one end of a microtubule is located in the B-cell zone and particularly the T-cell zone, while the other end is located in the red pulp or the marginal zone. With the presence of these microtubules in the spleen, the micro- molecular antigens can rapidly pass from the blood to the T-cell zone. Though these microtubules are distributed rarely in the B-cell zone, conduits may also serve as a pathway for low-molecular-weight antigens to reach follicles and facilitate B cell responses (Stranford and Ruddle, 2009).

## Directed Migration of Different Cells in the Spleen

The directed migration of different cell components is regulated by chemokines (**Table 1**), especially CCL21, CCL19, and CXCL13. The FRCs in the T-cell zone secrete CCL21 and CCL19, while after binding to the receptor CCR7 in T cells, they can chemoattract T cells (Ueha et al., 2011; Ugel et al., 2012). The FDCs in the B-cell zone secrete CXCL13, which functions by acting on its receptor CXCR5. CXCL13 guides B cells and follicular T helper (Th) cells into the B cell zone. The critical roles of these homeostatic chemokines in attracting cells into lymphoid organs and in initiating the antigen-specific responses are well-established (Mueller et al., 2007).

**Table 1:** Main chemokines and molecules in the spleen.

Chemokines and molecules	Receptor	Target cells	Expression regions	Promoting production	Inhibiting production	Producing cells	Function
CCL21	CCR7	T cells; DCs	T cell zone	LTs and TNFs	IFN-r	Mainly by FRCS	Attracting T cells
CCL19	CCR7	T cells; DCs	T cell zone	LTs and TNFs	IFN-r	Mainly by FRCS	Attracting T cells
CXCL13	CCR5	B cells; Th cells	B cell zone	LT-B	IFN-r	Mainly by FDCS	Attracting B cells
IL7	IL7R	T cells	T cell zone	LT-B	IFN-r and TGF-β1	Mainly by FRCS	T cell homeostasis
CCL2	CCR2	MDSCs	Margin zone; red pulp	Unknown	Unknown	Nestin^+^ splenocytes	Attracting MDSCs
S1P	S1PR1; S1PR3	B cells	Margin zone	Unknown	Unknown	LyVE-1^+^ cells	Attracting B cells
VCAM-1	VLA-4	HPSCs	Red pulp	Unknown	Unknown	VCAM-1^+^ macrophages	Attracting HPSCs

First, the different cell components in the blood will enter the marginal sinus through all levels of vascular branches, and thereby diffuse to the marginal zone. In this zone, the T and B cells will first adhere to the ER-TR7-positive fiberweb-like structure and migrate along the fasciculus in an oriented way. They first arrive at the marginal zone bridging channels (MZBCs) and then at the T-cell zone. The differentiation of T cells into follicular T helper (Th) cells will induce expression of CXCR5 in Th cells. Then Th cells and B cells would move further until arrive at the B-cell zone (Pereira et al., 2010).

Moreover, B cells express the chemokine receptor CXCR5 and thus can respond to the follicular attracting activity mediated by its ligand CXCL13. However, B cells could also overcome such activity by largely expressing the sphingosine-1 phosphate (S1P) receptors-1 and -3 (S1PR1, S1PR3). The binding of the lysophospholipid S1P which produced by LyVE-1^+^ cells to these receptors will trigger a chemotactic response and promote the accumulation of B cells in the marginal zone and red pulp which contains higher concentrations of S1P. Nevertheless, intravital microscopy studies indicate that MZBCs shuttle continually between the marginal zone and follicles. The mechanisms that control such oscillatory migration involves transient S1PR1 desensitization (for migration from the marginal zone into the follicles), followed by S1PR1 resensitization (for migration back to the marginal zone; Mehta et al., 2014). Moreover, the marginal zone is also permanently planted with many DCs, which, after digestion and reactivation with antigens, can express CCR7. Through the movement orbit of T cells, they could enter the T-cell zone and reactivate the antigen-specific T cells (Turley et al., 2010).

Just as mentioned before, except some kinds of cells (e.g., T cells, B cells, and DCs), other cells will flow along the blood stream into the red pulp. Myeloid-derived suppressor cells (MDSCs) which express CCR2 could be attracted by CCL2 and retain in the red pulp. Besides, bone marrow hematopoietic stem/progenitor cells (HSPCs) also could retain in the red pulp by vascular cell adhesion molecule 1 (VCAM-1)^+^ macrophages, which use the adhesion molecule VCAM-1 to retain HSPCs through its receptor – very late antigen 4 (VLA-4; Bronte and Pittet, 2013). In certain condition, a large amount of MDSCs or HSPCs could accumulate in the red pulp and influence the immune response. In the end, rest of the cells would flow out of the spleen along the blood stream (**Figure 1C**).

## Regulation of Chemokine Secretion

The expressions of CCL21 and CCL19 by FRCs in the splenic T cell zone and LNs facilitate the effective interaction between DCs and T cells (Luther et al., 2011). The secretion of these chemokines in SLOs is not constant, but can be regulated by different molecules. Firstly, previous research shows that the mesenchymal cells of SLOs require continuous signals from many molecules, so as to maintain their secretion of CCL21 and 19. The most-studied molecules are the tumor necrosis factor (TNF) family members. For example: LTs and TNFs are among such signaling molecules (Klaus et al., 1980; Schneider et al., 2004; Zhao et al., 2015). The deletion of these factors will induce the down-regulation of CCL21 and CCL19. However, the redundancy in TNF/LT ligand-receptor interactions complicates the understanding about the distinct contribution of particular ligand to the complex phenotype of TNF/LT-deficient mice (Tumanov et al., 2003; Zhao et al., 2014). In addition, these maintaining factors are mainly secreted by lymphocytes (Sixt et al., 2005; Zhao et al., 2014). As reported, the deletion of T cells will also lead to the significantly down-regulated secretory volumes of CCL19 and CCL21, and the expression levels of these chemokines can be recovered after the transfusion of T cells. Even the maintaining effects of these factors have not changed, some molecules also could directly down-regulate the expressions of CCL21 and CCL19. For instance, upon infection, the reactivated T cells will secrete abundant IFN-r, which will induce the down-regulated expressions of CCL21 and CCL19, and thereby down-regulate the immune response of T cells upon the next infection (Mueller et al., 2007; Li et al., 2014). In addition, expressions of CCL21 and CCL19 were also reduced in the spleen of Inflammatory Melanomas, but the reasons were not very clear (Soudja et al., 2011).

The chemokine CXCL13 is made broadly by follicular DCs (FDCs). Regulation of CXCL13 is much like the pattern of CCL21 and CCL19. Expression of CXCL13 is dependent on the cytokine lymphotoxin (LT)-B and a positive feedback loop involving CXCR5-mediated induction of LT-B expression by B cells contributes to maximal CXCL13 production and maturation of the FDC network. In addition, production of IFN-r also inhibits the expressions of CXCL13 which could inhibit B cells movement (Li et al., 2014).

Besides CCL21 and CCL19, the stromal cells FRCs can also secrete IL7 and participate in maintaining the homeostasis of T cells (Zhao et al., 2014). This secretion is mainly regulated by LT-B. The LT-B in the T-cell zone of SLOs is mainly secreted by T cells. Among acquired immune deficiency syndrome (AIDS) patients, the reduced count of T cells in SLOs will lead to the reduced secretion of LT-B and thus impact the secretion of IL7 (Sixt et al., 2005). Previous researches show that T regulatory cells could promote the procollagen production and subsequent deposition as fibrils via the TGF-β1 signaling pathway and chitinase 3-like-1 activity in fibroblasts in lymphoid tissues from human immunodeficiency virus (HIV)-1-infected patients. Collagen deposition restricts the access of T cell to the survival factor IL-7 on the FRC network, resulting in apoptosis and depletion of T cells, which in turn removes a major source of lymphotoxin-β (Fry and Mackall, 2005; Levy et al., 2009; Zeng et al., 2011, 2012).

Production of CCL2, which could induce a large amount of accumulation of MDSCs through CCR2, is strongly increased in tumor-bearing mice, and nestin^+^ splenocytes clearly participate in the increase in chemokine production in tumor- bearing hosts. These cells were rarely detectable in the spleen of tumor-free mice (Ugel et al., 2012).

## The Role of Blood Flow

Moreover, though not confirmed, we speculate that the blood flow rate in the spleen will also affect the speeds of different cell components entering the spleen. The reason is that when the expressions of various chemokines are unchanged, the blood flow rate will directly determine the speeds of different cell components entering the spleen, thereby impacting the speed of immune response. In clinic, the key factors that induce the changes of blood flow rate in the spleen are the blood pressure and the size of the spleen. When other conditions are not changed, the blood flow will change with the variation of blood pressure. When the blood pressure is unchanged, the increase of spleen size will accelerate the blood flow in the spleen. This may also explain why the spleen expands upon the onset of chronic infection in many patients. Therefore, the pathogen clearance rate can be improved by increasing the blood flow in the spleen.

## Immunosuppressive Cells

Myeloid-derived suppressor cells as immature myeloid cells are the precursors of DCs, macrophages, and/or granulocytes. Previous research shows that it is mainly distributed in the marginal zone and red pulp of the spleen (Ugel et al., 2012). The contents of immunosuppressive substances vary among MDSCs, with different populations containing arginase, inducible NO synthase, and/or additional ROS. Their accumulation has been documented in most patients and mice with cancer, which is induced by various factors produced by tumor cells and/or host cells in the tumor microenvironment. These substances will also accumulate in response to bacterial or parasitic infection, chemotherapy, experimentally induced autoimmunity, and stress. MDSCs are considered as a major contributor to the profound immune dysfunction of most patients with sizable tumor burdens (Ostrand-Rosenberg et al., 2012; Ugel et al., 2012). Previous research shows that these cells express CCR2, whose ligand is CCL2. Accumulation of MDSCs in the spleen could cause by increased production of CCL2 (**Table 2**). However, recent researches show that these cells also could proliferate in the spleen in certain condition. This also play an important role in the accumulation of these cells.

**Table 2:** Effects of chemokine disorders on splenic microenvironments.

Spleen region	Marginal zone		T cell zone		B cell zone		Red pulp	
	Chemokine change	Effect	Chemokine change	Effect	Chemokine change	Effect	Chemokine change	Effect
Infection	Unknown	Unknown	CCL21↓; CCL19↓	Inhibiting migration of T cells to T cell zone	CXCL13↓	Inhibiting migration of B cells to B cell zone	Unknown	Unknown
Tumor	CCL2↑	MDSCs accumulation	Unknown	Unknown	Unknown	Unknown	CCL2↑	MDSCs accumulation
Neonatal	Unknown	Unknown	Unknown	Unknown	Unknown	Unknown	Unknown	CD71^+^ erythroid progenitor cells accumulation
Anemia	Unknown	Unknown	Unknown	Unknown	Unknown	Unknown	Unknown	CD71+erythroid progenitor cells accumulation
HIV	Unknown	Unknown	IL7↓	T cells depletion	Unknown	Unknown	Unknown	Unknown
Inflammatory melanomas	CCL2↑	MDSCs accumulation	CCL21↓; CCL19↓	Inhibiting migration of T cells to T cell zone	Unknown	Unknown	CCL2↑	MDSCs accumulation

Moreover, as reported, bone marrow HSPCs can enter circulation in the steady-state. If conditions permit, the circulating HSPCs will produce lineage-descendant cells outside the bone marrow. This so-called extramedullary hematopoiesis occurs predominantly in the liver in the developing embryo, but also in adult tissues including the spleen. Red pulp vascular cell adhesion molecule 1 (VCAM-1)^+^ macrophages are essential to extramedullary myelopoiesis because these macrophages use the adhesion molecule VCAM-1 to retain HSPCs in the spleen red pulp. Indeed, under specific disease conditions, splenic HSPCs will profoundly expand. Splenic hematopoiesis has been reported in animal models of several diseases, including cancer, atherosclerosis myocardial infarction, and colitis (Cortez-Retamozo et al., 2012; Bronte and Pittet, 2013). However, there is no report whether these cells are active during immunoregulation. Nevertheless, recent research shows that CD71^+^ erythroid progenitor cells are physiologically enriched in neonatal mice, and CD71^+^ cells are produced after extensive proliferation and differentiation of HSPCs. These cells show immunosuppressive properties which could inhibit microphage function (**Table 2**). Specifically, the count of CD71^+^ erythroid cells in the spleens of new-born mice is significantly larger than that in mature mice. Moreover, Neonatal CD71^+^ cells express the enzyme arginase-2 and play important roles in the immunosuppression of new-born mice, which are different from mature mice (Elahi et al., 2013).

## Conclusion

The spleen undertakes important roles in SLOs, such as clearance of antigens from the blood, and generation of specific immune responses. Moreover, the spleen plays important roles in regulation of immune responses. Many functions of the spleen are unique and irreplaceable among other SLOs. Finally, more studies on the spleen are needed before dismissing its importance.

## Conflict of Interest Statement

The authors declare that the research was conducted in the absence of any commercial or financial relationships that could be construed as a potential conflict of interest.
